# Development of a School Nurse Data Science Boot Camp

**DOI:** 10.1177/1942602X261460808

**Published:** 2026-06-26

**Authors:** Jodi S. Bullard, Dixie Rose, Angela J. Preston, Janice Miles, Jenifer M. Chilton

**Affiliations:** Assistant Professor, School of Nursing, The University of Texas at Tyler, Tyler, TX, USA; Assistant Dean of Research/Assistant Professor, School of Nursing, Tyler, TX, USA; Assistant Professor, School of Nursing, The University of Texas at Tyler, Tyler, TX, USA; Clinical Associate Professor, School of Nursing, The University of Texas at Tyler, Tyler, TX, USA; Dean/Professor, School of Nursing, The University of Texas at Tyler, Tyler, TX, USA

**Keywords:** continuing education, professional development, school nurse education, School Nursing Practice Framework, systems-level leadership, leadership

## Abstract

A continuing education program was developed by School of Nursing faculty at a Northeast Texas university to strengthen school nurses’ ability to use data for effective communication. The 8‑hr *School Nurse Data Science Boot Camp* included a keynote speaker introducing data science concepts, followed by morning skill‑building stations on data interpretation and communication strategies. Afternoon stations guided participants in applying these skills to create slide presentations tailored to specific school nursing scenarios. The 15 participants were divided into five groups, each assigned a scenario and stakeholder audience. Groups developed and delivered presentations that demonstrated their ability to translate school health data into meaningful messages. Participants reported high satisfaction with the boot camp and expressed interest in an advanced offering. This program provides a valuable local continuing education opportunity and supports a growing regional partnership among school nurses, School of Nursing faculty, and Education Service Center personnel.

The school nursing environment is a rich repository of data that school nurses can use to advocate for improved health outcomes at the campus, school district, and community levels. Volumes of school nursing data are created daily through the documentation of health office visits, immunization records, screening mandates, and other sources. However, there is a gap in educational opportunities for school nurses to learn how to effectively use this data. The purpose of this manuscript is to describe an educational intervention created to promote school nurses becoming powerful users of their data. Leveraging data helps school nurses advocate for changes in their schools, provide education on health trends, and collaborate with others (National Association of School Nurses [NASN], 2019). Therefore, it is important to provide school nurses with educational opportunities to learn how to do this.

The collection and use of data by school nurses upholds the standards of school nursing practice as set forth in the four practice principles of the *School Nursing Practice Framework* (NASN, 2024a). These principles include (a) care coordination, (b) quality improvement, (c) leadership, and (d) community/public health. Implicit examples of data usage in care coordination include identifying patterns in the mental and physical health needs of students, recognizing social determinants of health impacting students and their families, and identifying progress in students’ care plans. Quality improvement explicitly states that school nurses should be engaged in data collection and analysis to benefit their patients and to self-reflect on their nursing practice. Implications of data usage in leadership include interpreting school health information, advocating for policies/procedures, and engaging in/influencing decision-making, all of which are strengthened by data. Community/public health involves the collection of data through student health screenings and immunizations while data analysis is necessary to identify student and community needs.

While the *School Nursing Practice Framework* clearly lays out the essential role of school nurses in data collection, analysis, and utilization, school nurses may require more training to feel confident in using their data as a communication tool. In situations where school nurses may be the only nurse on a campus or for an entire school district such as in rural areas, confidence in using their data when collaborating with educators and healthcare professionals in the community could play a significant role in their advocacy efforts for themselves and their patients.

## Background

Because of the work of Stanislo (2023), School of Nursing faculty at a Northeast Texas university were aware of how significant it is for school nurses to understand why collecting data is important, what data are beneficial, and how data can be accessed. These faculty members were also aware of limited participation in Texas, particularly by public school nurses and school districts, in the *Every Student Counts!* data collection initiative established by NASN (n.d.). With some of the faculty members having worked as school nurses in the region, they were aware that the rurality of the area (Texas Department of Agriculture, 2012) contributed to few school districts having a lead nurse in an administrative role able to focus on data. Because of these factors, they felt it was important to begin a dialogue with school nurses about the importance of their data. They were able to facilitate dialogue through their established community-academic partnership which provides the region’s school nurses with nursing continuing professional development (NCPD) opportunities.

### Initial Dialogue With School Nurses

School of Nursing faculty first introduced the topic of data at an annual conference for Northeast Texas school nurses in early July 2025. The presentation was titled *Using Data to Collaborate: Power in Numbers* (Bullard, 2025b) and focused on the value added to collaborative efforts by using data, and the responsibility of nurses to collaborate, as stated in the *Code of Ethics for Nurses* (American Nurses Association, 2025) and the *NASN Code of Ethics* (2024b). Throughout the presentation, Mentimeter, a personal device-driven classroom engagement tool, was utilized to facilitate audience engagement. Subsequently, the Mentimeter responses provided valuable insight regarding the school nurses’ perception of data.

**Figure 1.**
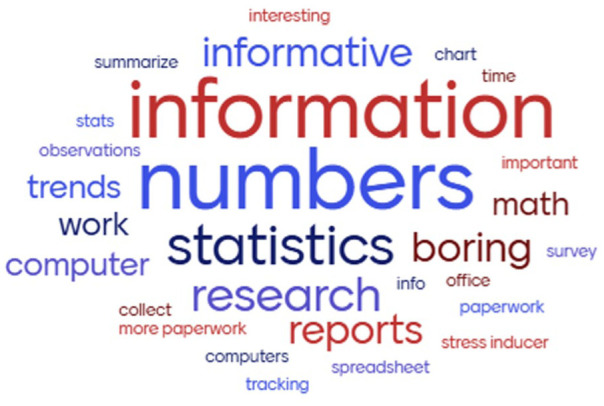
Mentimeter Word Cloud Results to the Question: In one word, what do you think when you hear the word “data”? *Source*. Bullard (2025b).

When asked through Mentimeter in a word cloud format “In one word, what do you think when you hear the word data?” Eighty-two school nurses responded using 28 different descriptors of data as seen in Figure 1 (Bullard, 2025b). With word cloud responses, larger font size words represent a greater number of people providing the response. “Numbers,” “Information,” and “Statistics” were the responses in the largest font size for this question. In addition to these responses, many other responses were related to data science such as “Research,” “Trends,” “Observations,” and “Tracking.” Other noteworthy perceptions of the word data were “Important,” “Interesting,” and “Informative” which were contrasted by the responses of “Boring,” “Stress Inducer,” and “More Paperwork.”

Some Mentimeter questions included a range of responses (*Never*, *Rarely*, *Sometimes*, *Often*, *Very Often*). For the question, “How often do you collect data as a school nurse?” 85 school nurses responded, with 35 responding *Very Often*, two responding *Rarely*, and the remaining responses being *Sometimes* and *Often*. When asked, “How often do you analyze data as a school nurse?” 86 school nurses responded, with five responding *Very Often*, three responding *Rarely*, and the remaining responses being *Sometimes* and *Often*. When asked “Who shows the most interest in your school nursing data?” response options included Lead Nurse or District Nurses, Principal or Assistant Principal(s), School Counselor(s), Front Office or Secretarial Staff, Superintendent or District Leadership, Teachers, and No One Else (Just Me!). For this question, 85 school nurses responded, and 53 stated their lead nurse/district nurses show the most interest in their data, while 9 stated that only they look at their data.

Participants were provided with the opportunity to offer feedback at the conclusion of the event regarding the *Using Data to Collaborate: Power in Numbers* presentation. In response to the question “Were the teaching methods/learner engagement strategies used by the presenter effective in meeting the learning outcomes?” of the 108 participants, 99% indicated that they were completely satisfied from a range of response options (*Not at all*, *Somewhat*, *Neutral*, *Almost Completely*, *Completely*). Additionally, 99% of participants indicated they would make changes to their practice/performance in areas such as “Data Collection,” “Use data to meet goals,” “Collaboration,” and “Documentation.”

### Second Dialogue With School Nurses

The second event where the topic of data was introduced was at a semiannual NCPD event for Northeast Texas school nurses held in late July 2025. The presentation was titled *Power in Data* (Bullard, 2025a) and utilized a case study to demonstrate the significance of using data to advocate and collaborate. Using data to communicate with facts was an overarching theme.

Participants were provided with the opportunity to offer feedback at the conclusion of the event regarding the *Power in Data* presentation. In response to the question, “Were the teaching methods/learner engagement strategies used by the presenter effective in meeting the learning outcomes,” of the 24 participants, 100% indicated that they were completely satisfied from a range of response options (*Not at all*, *Somewhat*, *Neutral*, *Almost Completely*, *Completely*). Additionally, 92% of participants indicated that they would be making changes to their practice/performance in areas such as “Documentation,” “Collaboration,” “Increase advocating for myself and students,” and “Data.”

## Identifying the Need for a Data Science Boot Camp

At the conclusion of the NCPD events, School of Nursing faculty reviewed the school nurses’ feedback. Noteworthy were responses to the question, “In one word, what do you think when you hear the word data?” in which some participants responded “Important,” “Interesting,” and “Informative,” in contrast to those who responded “Boring,” “Stress Inducer,” and “More Paperwork” indicating a wide range of opinions about data. Also, notable pertaining to the question “How often do you collect data as a school nurse?” was that less than half the school nurses reported collecting data very often, which was interesting considering that daily student health office visits involve the collection of data. Additionally, 35 nurses responded that they collect data very often, but only 5 nurses responded “Very Often” to the question “How often do you analyze data as a school nurse?” indicating a gap between school nurses’ data collection and data analysis. Finally, the question “Who shows the most interest in your school nursing data,” indicated that school nurses perceive that the primary interest in their data is centralized in the realm of nursing, with many saying either they or the lead nurse or other school district nurses show the most interest in their data.

Through the analysis of this information, School of Nursing faculty identified three areas where an educational event focused on data could benefit school nurses. First, such an event could fuel the enthusiasm of school nurses who view data through a positive lens while creating interest in those with greater hesitancy toward data usage. Second, further justification for the development of an educational event emanated from the school nurses’ responses to data collection and analysis. Providing school nurses with education on the wide array of data they collect in the school clinic could be valuable in increasing their interest in using their data. Lastly, based on the perception of school nurses that the main interest in their data is within the silo of school nursing, providing education on the benefit of school nursing data to other disciplines could improve interdisciplinary collaboration. For these reasons, the School of Nursing faculty determined there was sufficient evidence to support the creation of the *School Nurse Data Science Boot Camp*.

## Development and Implementation of the School Nurse Data Science Boot Camp

The overall framework for the data science boot camp was modeled after an NCPD event designed to fill a gap for continuing education opportunities and provide a record of competency for school nurses (Bullard et al., 2025). An important consideration in developing the data science boot camp was consulting with the NCPD team in the School of Nursing to ensure that continuing education credits could be offered for the event. Another consideration was access to computers for the participants to utilize the internet to search for best practice literature, Microsoft Excel for data analysis and data visualization, and Microsoft PowerPoint to develop a presentation. The School of Nursing data science lab at the university could meet these needs and accommodate 20 participants. An additional consideration was working with the community partner, the regional Education Service Center, regarding participant registration and payment collection.

**Figure 2.**
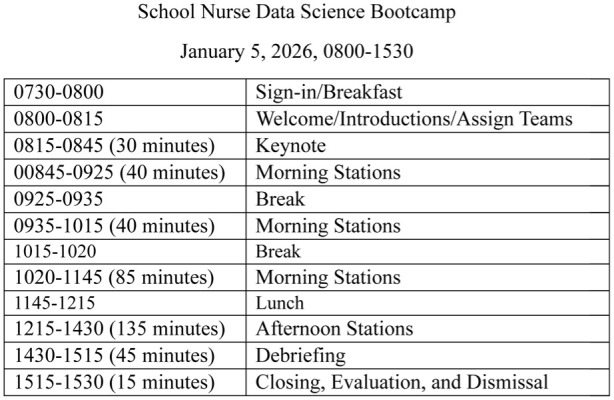
Example Agenda

Once event location and acceptability to facilitate registration/payment by the Education Service Center were confirmed, January 5, 2026, was selected as the event date to accommodate the work calendars of most of the region’s school nurses whose school districts scheduled staff development on that day. The data science boot camp was designed to be a 1-day, 8-hr event beginning with a keynote address about data science followed by morning stations, lunch, afternoon stations, and an afternoon debriefing period (see Figure 2).

At the beginning of the day, participants were divided into five teams, and each team was assigned one of five scenarios. The scenarios addressed a range of school health issues that school nurses may need to bring forward for discussion in their school districts. Topics included proposed health policy changes, the need for interdepartmental collaboration, a communicable disease concern, justification for maintaining an existing health policy, and a clinic traffic-management concern.

Instructors of the morning stations provided training on enhancing communication with credible sources, communicating effectively with decision-makers, and Microsoft Excel basics including navigating data, data analysis, descriptive statistics, and data visualization (tables and graphs). Integrated into the stations were opportunities for each group to discuss and complete hands-on activities using their scenario. As a result of these activities, at the end of the morning every group had discussed and prepared materials needed for the afternoon stations.

The afternoon stations involved the development of a slideshow by each group, followed by a mock presentation to the specified stakeholder from each scenario. Scenario stakeholders included a school board, a superintendent, a principal, an athletic director, and a school district’s faculty/staff. Boot camp participants served as the stakeholder audience for each other’s presentations.

Prior to beginning work on the presentation, participants received instruction on slideshow development. They were asked to create a 3-to-5-min slideshow to effectively communicate the need for change to their assigned stakeholder. Slides were to include an introduction to the topic/problem, literature supportive of change, Microsoft Excel data analysis results/graphics to substantiate the proposed change, and a synthesis of how the presented information supported their recommendation.

The event concluded with a debriefing period where participants shared insights into their boot camp experience. The intent of the boot camp was to fuel school nurses’ enthusiasm toward data, highlight the wide array of data they collect, and get them thinking of how they can use their data to interact with others outside of the school nursing silo. Feedback from the debriefing was enthusiastic, with participants requesting an advanced data science boot camp focused on more in-depth aspects of data analytics and visualization. Overall, participants indicated that the School Nurse Data Science Boot Camp provided them with skills to become powerful users of their data, supporting them in carrying out their role as leaders, care coordinators, and advocates for positive evidence-based change in the school setting.

## Conclusion

The *NASN School Nursing Practice Framework* outlines the importance of school nurses in data collection, analysis, and utilization, and the School Nurse Data Science Bootcamp provides a tangible and pragmatically useful opportunity to advance school nurse data usage. The academic partnership between School of Nursing Faculty of the Northeast Texas University and the regional Education Service Center serves as an exemplar for developing school nurses’ knowledge of data science and how to use data to advocate for themselves and students. The role of technology in society is continuing to increase, making it important that school nurses are equipped to utilize data to address population-level health trends, engage in school nursing research, and contribute as active members of the interdisciplinary school health service delivery teams.

## References

[bibr1-1942602X261460808] American Nurses Association. (2025). Code of ethics for nurses. https://codeofethics.ana.org/home

[bibr2-1942602X261460808] Bullard J. S. (2025a, July 28). Power in data [Conference session]. School Nurse Simulation Boot Camp, Tyler, TX, United States.

[bibr3-1942602X261460808] Bullard J. S. (2025b, July 9). Using data to collaborate: Power in numbers [Conference session]. 2025 Hot Topics for School Nurses: Region 7 Education Service Center School Nurse Annual Conference, Tyler, TX, United States.

[bibr4-1942602X261460808] Bullard J. S. Preston A. J. Chilton J. M. (2025). Development of a school nurse simulation boot camp. NASN School Nurse, 40(5), 248–252. 10.1177/1942602X251350104 PMC12426319 40618245

[bibr5-1942602X261460808] National Association of School Nurses. (n.d.). National school health data set: Every student counts! https://www.nasn.org/research/everystudentcounts

[bibr6-1942602X261460808] National Association of School Nurses. (2019). Share to make aware: Ten ways to share your data. https://higherlogicdownload.s3.amazonaws.com/NASN/8575d1b7-94ad-45ab-808e-d45019cc5c08/UploadedImages/PDFs/Research/Data_10_ways_to_share_your_data.pdf

[bibr7-1942602X261460808] National Association of School Nurses. (2024a). A contemporary framework update for today’s school nursing landscape: Introducing the School Nursing Practice Framework. NASN School Nurse, 39(3), 140–147. 10.1177/1942602X241241092 38623932

[bibr8-1942602X261460808] National Association of School Nurses. (2024b, June). Code of ethics. https://www.nasn.org/nasn-resources/codeofethics

[bibr9-1942602X261460808] Stanislo K. J. (2023). Data collection: Time to revisit the WHY, WHAT, and HOW. NASN School Nurse, 38(6), 310–315. 10.1177/1942602X231199932 37735899

[bibr10-1942602X261460808] Texas Department of Agriculture. (2012). Texas county designations. https://texasagriculture.gov/portals/0/forms/er/rural-metro%20counties.pdf

